# Exploring barriers to accessing physiotherapy services for stroke patients at Tema general hospital, Ghana

**DOI:** 10.1186/s40945-017-0037-5

**Published:** 2017-07-06

**Authors:** Mercy Nketia-Kyere, Genevieve Cecilia Aryeetey, Justice Nonvignon, Moses Aikins

**Affiliations:** 1 Tema General Hospital, Tema, Ghana; 2 Department of Health Policy, Planning and Management, School of Public Health, College of Health Sciences, University of Ghana, P. O. Box LG13, Legon, Accra, Ghana

**Keywords:** Physiotherapy, Stroke, Barriers, Ghana

## Abstract

**Background:**

Physiotherapy has been shown to reduce the risk of disability among stroke patients. Poor adherence to physiotherapy can negatively affect outcomes and healthcare cost. However, very little is known about barriers especially to physiotherapy services in Ghana. The objective of this study was to assess the barriers to physiotherapy services for stroke patients at Tema General Hospital (TGH). The individual/personal and health system barriers to physiotherapy services at TGH were determined.

**Method:**

A cross-sectional study design was employed. A simple random sampling technique was used to recruit 207 respondents for a face-to-face interview. Interviewer-administered questionnaires were used to collect data on individual/personal barriers of respondents to physiotherapy services and were described using the Likert’s scale. Health system barriers were assessed using a self-structured questionnaire which had section under the following heading: human factors, physiotherapy modalities, physical barriers and material/equipment factors. The time spent waiting for physiotherapy and attitude of physiotherapist towards patients; physiotherapy modality such as electrotherapy, exercise therapy and massage therapy among others were some of the indices measured. Respondents’ adherence to Medication was assessed with the Morisky 8-item medication adherence questionnaire. Data were entered and analysed using Epi info 7 and STATA 12.0. Associations between the variables were determined using a chi-square test and logistic regression model was used to test the strength of associations between the independent and the dependent variables. The level of statistical significance was set at *p* < 0.05.

**Results:**

The results showed that majority (76.3%) of the respondents had economic barrier as their main individual/personal barrier to physiotherapy services. For medication adherence level, patients with low medication adherence level were about 21 times the odds of defaulting on accessing physiotherapy services five times or more as compared to those with medium adherence level (OR 20.63, 95% CI 8.96, 42.97). It was concluded in the study that individual/personal barriers of stroke patients were the significant barriers to accessing physiotherapy services at Tema General Hospital.

**Electronic supplementary material:**

The online version of this article (doi:10.1186/s40945-017-0037-5) contains supplementary material, which is available to authorized users.

## Background

Stroke or cerebrovascular accident is a common brain disorder characterised by sudden neurological symptoms, such as paralysis or loss of sensation resulting from the destruction of brain tissue, intracerebral haemorrhage, emboli and atherosclerosis of the cerebral arteries [1]. Indeed stroke is a global health problem identified as the second commonest cause of death and leading cause of adult disability [2, 3]. The burden of stroke is expected to increase in the next 20 years as the global population ages. Without any intervention, Strong [4] argued that global deaths from stroke are likely to increase to about 7.8 million by 2030. Developing countries have recorded the highest mortality from stroke, accounting for more than two-thirds of global deaths from this condition [2].

In Ghana, stroke has been found to be a major contributor to disability and reduced quality of life [5], with more than half of all stroke survivors left dependent on others for everyday activities [6]. The prevalence of the disease is on the increase and this is attributed to lifestyle changes and unhealthy dietary practices [7]. Many facilities now report increase in number of patients reporting for physiotherapy [Tema General Hospital, Unpublished Observations]. It has been found that, physiotherapy improves the lives of the patients by reducing pain and preventing complications and improving general quality of life [8]. A number of studies have also suggested that rehabilitation programmes lead to an improvement in functional status that cannot be attributed merely to spontaneous recovery [9]. Subsequently, the Ministry of Health (MoH) has introduced physiotherapy departments in most major public hospitals throughout the country, Tema General Hospital (TGH) is one of them [10]. The target participants for this study is stroke patients attending the Tema General Hospital (TGH) Physiotherapy department. Reports from the department showed that 25% and 35% of patients who attended the hospital in 2011 and 2012 were patients suffering from stroke. Nonetheless, the default rates for physiotherapy sessions for these patients were equally high i.e. patients were usually unable to complete the eighteen recommended number of sessions for physiotherapy [Tema General Hospital, Unpublished Observations]. The non-adherence of physiotherapy was found to negatively affect the outcomes and cost of healthcare [11]. Some identified factors such as, the individual undergoing the treatment, the kind of facilities available, the quality of staff managing the facilities and quality of medication available could be contributing to the success or failure of treatment regimen undertaken by stroke patients. Unfortunately, the actual effect of these variables on physiotherapy treatment of stroke patients is not yet known [11].

This study seeks to assess the barriers to physiotherapy services for stroke patients. It thus, seeks to provide useful information to hospital management on how to tackle most of these barriers and to enhance adherence to physiotherapy services to achieve better outcome for stroke patients and ensure effective and efficient management of stroke patients in Ghana. The study therefore sought to answer the following questions; what are the individual barriers to physiotherapy services for stroke patients at TGH? What are the health system barriers to physiotherapy services for stroke patients at TGH?

### Conceptual framework

**Fig. 1:**
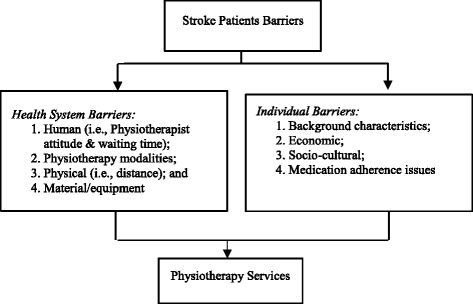
Conceptual framework of barriers to physiotherapy services for stroke patients

The barriers to physiotherapy services can be discussed under two main categories, namely individual/personal and health system barriers. The individual/personal barriers are the barriers that pertain to the stroke patients, comprising of background characteristics, economic, socio-cultural, medication barriers and medication adherence levels. The health system barriers are those barriers that affect the service delivery system; it includes; human (physiotherapists’ attitude), physiotherapy modalities (exercise therapy, thermotherapy, electrotherapy, cryotherapy, massage therapy, among others) waiting time, physical barriers (distance to the physiotherapy facility and wheelchair/walking aid accessibility) and material/equipment [12], as illustrated in Fig. 1.

## Methods

### Study design

A cross-sectional study was undertaken in June 2015 to examine barriers to physiotherapy treatment by stroke patients.

### Study setting and sampling

The study was undertaken at the Physiotherapy Department of the Tema General Hospital in Ghana. The hospital provides physiotherapy services to communities within the Tema metropolis and beyond. The physiotherapy department has a patient turn out rate of 80% of which 40% are persons who suffer from stroke. The department reserves 5 working days a week to serve stroke patients.

A random sampling technique was used to select stroke patients from the attendance register. Next, the folders of stroke patients who had not completed their therapy or had defaulted two consecutive or more sessions/visits for the past six months were selected. A total of 430 stroke patients were obtained as eligible study participants. This served as the sampling frame. The folders were numbered/listed from 1 to 430. We used a computer generated random numbers to select the estimated sample size of 216 participants. From the 216 identified participants, we selected patients who are older than 18, conscious, with good memory and capable of giving voluntary consent.

### Data collection

We collected data on patients with good memory and able to give inform consent. A face-to-face interview technique was used for data collection. Each interview utilised a structured questionnaire, and lasted for about thirty (30) minutes. The questionnaire covered information on stroke patients’ demographic characteristics, socio-cultural, economic, medication and health system barriers. Physical factors such as accessibility to wheelchair were assessed using Likert’s scale. The Morisky 8-item Medication Adherence level scores were classified as zero (0) being high adherence, one (1) and two (2) as medium adherence and more than two (>2) as low adherence. The Morisky 8-item Medication Adherence Questionnaire was used to measure medication adherence level of the stroke patients (Additional file 1). The Pearson’s correlation coefficient was used to concurrently validate the scale with a previously validated 4-item measure of adherence [11]. The reliability of the 8-item scale was determined using standard statistical procedures described by Cronbach [11]. The validity of the questionnaire was determined through a pre-test carried out at the Ridge Hospital using stroke patients at the physiotherapy department of Ridge Hospital. This was done to test the self-develop questionnaire. The inclusion criteria were confirmed from the records at the Physiotherapy Department of TGH and also the patients were asked follow up question to confirm that they fell into the category of stroke patients that were needed for the study.

Patients were not on admission but were visiting the physiotherapy department of the Tema General Hospital for physiotherapy services on Out Patient Department (OPD) basis. The inclusion criteria were stroke patients 18 years and above, conscious and had good memory, in good state of mind and were capable of giving voluntary consent. All stroke patients who had reported and received services at the Department for the past 6 months and had defaulted for two consecutive times or more in 1 week were selected as potential respondents. Stroke patients who were on two and three physiotherapy session programme per week were selected. Patients who have defaulted or stopped receiving therapy as a result of ill-health were excluded. A selected patient who did not qualify within the inclusion criteria was skipped and the next qualified patient in the list (sample frame) was selected and interviewed. English language was used to interview the respondents who understood or spoke English whiles for those who did not understand English, the questionnaire was translated in their native languages and their response recorded on the questionnaire in the English language.

### Data analysis

Patients were classified into low and high defaulters. Low defaulters were patients who missed physiotherapy sessions less than 5 times out of the 18 sessions required, whilst high defaulters were patients who have missed 5 or more sessions.

Descriptive statistics were used to analyse demographic characteristics of respondents. Two main barriers were identified; Individual and health systems barriers. Individual barriers include socio-cultural, economic, medication and adherence barriers. Health systems barriers include attitude of health professionals and availability of equipment for use at the facility. A Pearson’s Chi square test was carried out to identify the associations between the study barriers and the rate of default. A logistic regression analysis was used to test the strength of association between the study barriers.

## Results

A total of 207 patients participated in the study. About 57% were males and 42% females. About 10% of the patients were below 40 years, 24% with secondary level education, 73% married and 54% still in employment.

### Main barriers

#### Socio-cultural barriers

Most of the respondents 62 (30%) had no socio-cultural barrier. Twenty-eight percent of them had the belief that treatment of stroke was spiritual. Current physical state (27.5%) was noted as a barrier to assessing physiotherapy services. Family (9.2%) and friends (5.3%) support were minimal. There was no significant association between the socio-cultural barriers and the rate of default (χ2 = 4.13; *p* < 0.39).

#### Economic barriers

About 92 (44.4%) of the stroke patients identified financial dependence and transportation cost (23.7%, *n* = 49) as their main barriers. Only a small proportion of patients cited physiotherapy services cost (8.2%, *n* = 17) and payment of caregivers services (7.3%, *n* = 15) as economic barriers. However, 34 (16.4%) of the respondents did not identify any economic barrier. There was no significant association between the economic barriers and the rate of default (χ2 = 3.82; *p* < 0.43).

#### Medication barriers

Medication cost (41.6%, *n* = 86), medication side effect (22.7%, *n* = 47) and medication adherence (21.3%, *n* = 44), were identified as barrier to physiotherapy services. There was a significant association between the patients medication barriers and the rate of default (χ2 = 16.18; *p* < 0.001).

#### Medication adherence level

Most of the patients (58.9%, *n* = 122) had low medication adherence level. There was a significant association between the patients’ level of adherence to medication and the rate of default (χ2 = 84.54; *p* < 0.001).

**Fig. 2:**
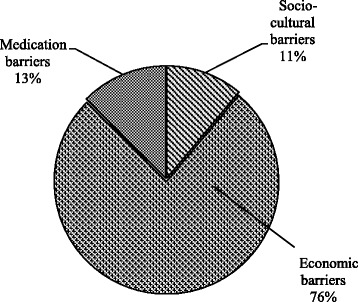
Proportion of main individual barriers of respondents

Economic barrier (76.3%, *n* = 158) was identified as patients’ main individual barrier to physiotherapy services. This was followed by medication barrier (12.6%, *n* = 26), and socio-cultural barrier (11.1%, *n* = 23) as indicated in Fig. 2.

### Association between barriers and default on physiotherapy services

Among the low defaulters, there were more men (54.8%) compared to women (45.2%). Of this group, a third (31.7%) were aged 50–59 years, a third (31.7%) had secondary education, most (70.2%) were married and about 55.8% were unemployed. Generally, among the high defaulters, there were more men (60.3%) compared to women (39.8%). Of this group, about a third (28.2) % were aged 50–59 years, over a third (36.9%) had tertiary education, majority (76.7%) were married and 65.2% were employed. There was a significant association between employment status and default (**χ** ^**2**^ = 9.05; *p* = 0.003) as well as education (**χ** ^**2**^ = 12.18; *p* = 0.016).

Among the low defaulters, over a third (33.7%) had no socio-cultural barrier. About 40% stated financial dependence as an economic barrier, 50% also stated medication cost as a barrier while majority of them (71.2%) had medium level of medication adherence. For the high defaulters, about 30% had beliefs/spiritual treatment for stroke as a socio-cultural barrier. About 49% stated financial dependence as an economic barrier, 33% also stated medication cost as a barrier and majority of them (89.3%) had low level of medication adherence. There was a significant association between the patients medication barriers (**χ** ^**2**^ = 16.18; *p* < 0.001), medication adherence level (**χ** ^**2**^ = 84.54; *p* < 0.001) and default rate.

Participants who disagreed that the physiotherapists were not friendly had high default rate of 89 (86.4%), whilst majority of participants who also agreed that physiotherapy facility was wheelchair/walking aid friendly showed a high default rate of (70.9%). However, there was no significant association between the rate of default and physiotherapists’ friendliness (χ2 = 0.108; *p* < 0.947) and the rate of default and the facility being wheelchair friendly as well (χ2 = 0.4618; *p* < 0.794).

### Individual barriers influencing physiotherapy services

Table 1 shows the association between some individual barriers and physiotherapy services. For educational level, those with primary education were eight times more likely of defaulting on physiotherapy services 5 or more times as compared to those with no education (OR 7.5, 95% CI 1.31, 43.03). Employed patients were 3 times more likely to default on physiotherapy services 5 or more times (OR 2.35, 95% CI 1.34, 4.11) compared to those unemployed. On medication, medication adherence had about 4 times odds of defaulting on physiotherapy services 5 or more times as compared to the medication cost. Patients with low adherence level to medication had about 21 odds of defaulting on physiotherapy services 5 or more times compared to those with medium adherence level. These results were all statistically significant (*p* < 0.05 and *p* < 0.001).

**Table 1:** Association between individual barriers and default on physiotherapy services

	Crude		Adjusted	
Variable	OR (95% CI)	*P*-value	OR (95% CI)	*P*-value
Age (years):		0.303		
<40	Ref			
40–49	0.94 (0.32; 2.75)			
50–59	0.54 (0.19; 1.49)			
60–69	0.47 (0.16; 1.32)			
70+	0.47 (0.15; 1.47)			
Sex:		0.433		
Male	Ref			
Female	0.80 (0.46, 1.39)			
Marital status:		0.553		
Married	Ref			
Single	1.04(0.38, 2.84)			
Widowed	0.55(0.23, 1.34)			
Divorced	0.69 (0.23, 2.09)			
Current level of education:		0.014*		0.265
No education	Ref		Ref	
Primary	7.50(1.31, 43.03)		1.64(0.20,13.45)	
Middle/JHS	4.02(1.01, 16.01)		2.34(0.38,14.34)	
Secondary/Vocational	1.82(0.44, 7.46)		1.07 (0.17, 6.84)	
Tertiary	4.52 (1.14,17.97)		3.08 (0.48, 19.90)	
Employment status:		0.002*		0.007*
Not employed	Ref		Ref	
Employed	2.35 (1.34, 4.11)		2.87(1.32, 6.22)	
Main socio-cultural barriers:	Ref	0.380		
Beliefs/spiritual treatment	1.89(0.63,5.65)			
Lack of family support	0.73(0.20,2.65)			
Lack of friends’ support	0.78(0.38,1.63)			
People seeing me in condition	0.67(0.33, 1.38)			
None				
Main economic barriers:				
Affordability of services	Ref	0.418		
Affordability of transport	2.12 (0.65, 6.94)			
Financial dependence	2.86 (0.93, 8.77)			
Hiring a caregiver	2.74 (0.64,11.75)			
None	2.40 (0.15,1.18)			
Medication barrier:		<0.001**		0.876
Medication cost	Ref		Ref	
Adherence	3.65 (1.67,7.95)		1.05(0.39, 2.83)	
Side effects	2.25 (1.09,4.66)		0.70(0.25, 1.94)	
None	2.74 (0.32,1.83)		1.01(0.32, 3.17)	
Adherence level:		<0.001**		<0.001**
Medium	Ref		Ref	
Low	20.63(8.96,42.97)		22.07(8.8, 55.37)	

*P*-value <0.05* *P*-value <0.001**

### Health system barriers influencing physiotherapy services

There was no significant association between the various health system barriers and default on accessing physiotherapy services. Those who agreed that the physiotherapists were not assisting them to go through procedures had 1.5 times odds more likely of defaulting 5 or more times than those who disagreed (OR 1.50, 95% CI: 0.54, 4.08). The association between the default to physiotherapy services and physiotherapists not assisting showed no statistical significance (*p* = 0.434). Waiting time (*p* = 0.109), and physiotherapists’ friendliness (*p* = 0.379) were also not found to be significantly associated to default. On the responses for the difficult modalities, those who had no difficulty with the modalities had 2.3 odds more of defaulting 5 times or more as compared to those who opined that exercise therapy was the difficult modality (OR 2.2, 95% CI: 60.95, 5.34).

## Discussion

In this study, the patients were predominantly males, between the ages 50–59, having tertiary education and mostly married. This is consistent with the findings of Donkor et al., [13] who assessed the epidemiology, quality of life and community perceptions of stroke patients in southern Ghana. A study by WHO [14] on Ghana Country Assessment Report on ageing and health also showed similar age distribution of stroke patients. In that study male patients were more affected than their female counterparts. This may be related to the fact that males are more prone to the risk factors associated with stroke, especially, those concerned with poor lifestyle such as smoking and drinking compared to females. This can be compared with the study by Urimubenshi and Rhoda [15] who found that more than half of the respondents affected by stroke were males.

This study also found that over 90% of the respondents had at least primary education. This is comparatively higher than the 50% reported by Urimubenshi and Rhoda [15]. This can be explained partly by the fact that the setting for this study is made up of people predominantly in the upper and middle class and are more likely to have had at least primary education. Interestingly, stroke patients with tertiary education had high default rate. Again, the study showed that those with education had higher odds of defaulting than those with no education. This could be attributed to the fact that, this group of patients were more likely to be financially sound and hence more likely to try alternative remedies. Additionally, this group may have had knowledge of other alternative treatment available. It could also be explained by the fact that those with tertiary education (who were capable of going to work) have high tendency of being employed in better jobs which are likely to be time demanding thus limiting their time for physiotherapy sessions. Additionally, the setting where the study took place had a number of alternative treatments available and patients would be more likely to utilise these services for remedy, consequently, leading to their high default of physiotherapy services at TGH.

On socio-cultural barriers, about one-third of the stroke patients reported having problem with people seeing them in their conditions. Subsequently, this served to be a barrier against their adherence to physiotherapy. This may be suggestive of poor social attitude towards stroke. Similarly, most people were of the belief that stroke has a spiritual cause and hence would require other remedies other than physiotherapy. Consequently, this served to be the main socio-cultural barrier that affected the rate of default to physiotherapy services for stroke patients. Accordingly, Payne [16] suggested that cultural beliefs should be considered in providing care to people with stroke. A substantial number (30%) of the patients in this study reported having no challenge with regards to socio-cultural barriers to physiotherapy. This could be explained by the fact that most of the patients in this study had high level of education. A study by Coulter et al., [17] showed that level of education improves the perception of patients as well as their general beliefs.

Economic barrier was the main individual barrier to physiotherapy sessions. This may be related to the fact that each individual had different levels of financial commitments to the condition. According to McNamara, Normand and Whelan [18] the living status of a person affects his/her use of primary care and some hospital services including physiotherapy care. In this research, medication cost appeared to be the main barrier to medication treatment of stroke patients who visit the TGH. This could have resulted also from the fact that a significant proportion of the patients were unemployed and thus do not have much available or disposable income. The current results are in line with previous observations by Stuart and Zacker [19] who found drug costs to be a barrier to adherence to drug therapy by stroke patients. A study by Munger et al., [20] suggested that duration of treatment with medication influences the rate of non-adherence to medication by stroke patients. The chi-squared test showed that the various barriers to medication treatment of the stroke patients were affected by the level of education. Most of the stroke patients at the TGH had low adherence level to medication, which could have also influenced the rate of default. This could be explained by the fact that the respondents were receiving alternative form of treatment and did not adhere to their medications. In general, this study found the individual barriers such as educational level, employment status, medication barriers and medication adherence levels to be the most contributed to defaulting on accessing physiotherapy services.

We found that attitude of physiotherapists did not serve as a barrier to default on accessing physiotherapy services. Physiotherapists were generally considered to be friendly and supportive to stroke patients during physiotherapy procedures. The warm reception and responsiveness exhibited towards stroke patients by physiotherapists could have encouraged many patients to patronise physiotherapy services. The majority of stroke patients attested receiving various assistance from physiotherapists, during therapy sessions. The supports could have been in the form of assisting or supporting patients during exercise therapy and other modalities. This is consistent with findings of Jack et al., [11] who reported that physiotherapists’ attitude if poor can be a barrier. The receptive attitude of the staff towards the patients can be as a result of the fact that they had adequate skills. This is in line with Langhorne et al., [21] who suggested that an adequate number of skilled staff is an essential requirement of stroke-unit care. Thus, various skills will be needed for delivery of adequate care. This is however in contrast with studies by Lacy et al., [22] who reported on issues of respect by the health personnel and health care staff who did not respect patients, their opinions and emotions.

With regards to physical barriers, most of the stroke patients agreed that the facility was wheelchair/walking aid friendly. This is however contrary to the findings of Urimubenshi and Rhoda [15] who reported that most of the participants had challenges with regards to accessibility to physiotherapy facility in the Musanze district of Rwanda. Studies by Mazie’res et al., [23] showed that factors associated with adherence to physiotherapy are easy access to a facility where one performed exercises. In general, this study revealed that health system barriers did not contribute to the default on accessing physiotherapy services for stroke patients.

## Conclusion

The study sought to assess the barriers to physiotherapy services for stroke patients at Tema General Hospital. Individual and health system barriers to physiotherapy services for stroke patients at Tema General Hospital were assessed. The study found individual barriers such as employment status, current educational level, medication barriers and medication adherence level to be the significant barriers that contributed to default on accessing physiotherapy services for stroke patients at TGH. However, none of the health system barriers contributed significantly to default on accessing physiotherapy services for stroke patients at Tema General Hospital.

## Additional file

Additional file 1: Structured questionnaire. (DOCX 23 kb)

## Abbreviations

GHS-ERC: Ghana Health Service Ethical Review Committee
MoH: Ministry of Health
TGH: Tema General Hospital
