# Supplementary material for: Exploring barriers to accessing physiotherapy services for stroke patients at Tema general hospital, Ghana
Source: Arch Physiother. 2017 Jul 6;7:8. doi: 10.1186/s40945-017-0037-5 (PMC5759901; doi:10.1186/s40945-017-0037-5)
Supplement: Additional file 1: — Structured questionnaire. (DOCX 23 kb) [file 40945_2017_37_MOESM1_ESM.docx]

**Questionnaire**

**Date: ___________________**

Dear Respondent,

This is a research carried out on assessment of barriers to physiotherapy services for stroke patients in Tema General Hospital. I will therefore like you to take a little time with you to answer these questions. You are assured that the answers you will give will be strictly confidential and would not be held against you.

**SECTION A: STRUCTURED QUESTIONNAIRE.**

| **Number** | **Question** | **Response.** |
| --- | --- | --- |
|  | Respondents ID | \|  \|  \|  \| \| --- \| --- \| --- \| |
|  | Demographic Information |  |
| 1 | Sex  1.Male  2. Female | \|  \| \| --- \| |
| 2 | Age | \|  \|  \| \| --- \| --- \| |
| 3 | What is your current level of education?  1. No education.  2. Primary  3. Middle/JHS  4. Secondary/Vocational.  5.Tertiary | \|  \| \| --- \| |
| 4. | What is your marital status?  1.Married  2.Not married  3. Widowed  4. Divorced | \|  \| \| --- \| |
| 5. | Employment status  1.Unemployed  2. Employed | \|  \| \| --- \| |
| **A** | **HEALTH SYSTEM BARRIERS** |  |
| **(i)** | **Physiotherapists’ Attitude** |  |
| 6. | Some physiotherapists are not friendly.   1. Strongly disagree 2. Disagree 3. Unsure 4. Agree 5. Strongly agree | \|  \| \| --- \| |
| 7 | The therapist doesn’t assist me to go through procedures.   1. Strongly disagree 2. Disagree 3. Unsure. 4. Agree 5. Strongly agree | \|  \| \| --- \| |
| **(ii)** | **Waiting Time** |  |
| 8 | I wait for too long before I receive therapy.   1. Strongly disagree 2. Disagree 3. Unsure. 4. Agree 5. Strongly agree | \|  \| \| --- \| |
| **(iii)** | **Physiotherapy Modality** |  |
| 9. | Which of the following modalities is very difficult for you to undertake?   1. Exercise therapy 2. Electrotherapy 3. Massage therapy 4. Thermo/heat therapy 5. Cryotherapy 6. Other (Specify) | \|  \| \| --- \| |
| 10. | How many modalities do you go through per therapy session?   1. One 2. Two 3. Three 4. Four | \|  \| \| --- \| |
| **iv)** | **Physical Barriers** |  |
| –11 | The physiotherapy facility is wheelchair/ walking aid friendly.   1. Strongly disagree 2. Disagree 3. Unsure. 4. Agree 5. Strongly agree | \|  \| \| --- \| |
|  | **Distance to Physiotherapy Centre** |  |
| 12. | My house is far from the physiotherapy department.   1. Strongly disagree 2. Disagree 3. Unsure. 4. Agree 5. Strongly agree | \|  \| \| --- \| |
| 13. | How many minutes does it take you to come to the physiotherapy department? | ______________Minutes |
| **(vi)** | **Material/Equipment** |  |
| 14. | There is enough equipment available to use for my therapy at the physiotherapy department?   1. Strongly disagree 2. Disagree 3. Unsure. 4. Agree 5. Strongly agree | \|  \| \| --- \| |
| **B** | **PERSONAL/INDIVIDUAL BARRIERS** |  |
| **i** | **Socio-cultural barriers** |  |
| 15. | What is your main socio-cultural barrier to physiotherapy services   1. Belief/spiritual treatment for stroke 2. Lack of family support 3. Lack of support from friends. 4. People seeing me in this condition 5. None 6. Other(Specify)_____________________ | \|  \| \| --- \| |
| **ii** | **Economic barriers** |  |
| 16. | What is your main economic barrier to physiotherapy services?   1. Affordability of physiotherapy Service 2. Affordability of transport 3. Financial dependence/ not working 4. Payment/Hiring a caregiver 5. None 6. Other (Specify)________________ | \|  \| \| --- \| |
| **iii** | **Medication Barriers** |  |
| 17. | What is your medication barrier to physiotherapy services?   1. Medication cost 2. Adherence 3. Side effects 4. Other (Specify)____________________ | \|  \| \| --- \| |
| 18. | Of all these individual barriers to physiotherapy services, which one is the main barrier to you?   1. Socio-cultural barriers 2. Economic barriers 3. Medication barriers. | \|  \| \| --- \| |

**SECTION B. MORISKY 8 ITEM MEDICATION ADHERENCE QUESTIONNAIRE**

Patients answer Yes (Y) = 1; No (N) = 0

| **Number** | **Morisky Medication Adherence**  **Scale-8(MMAS-8). Question.** | **Response** | | **Codes** |
| --- | --- | --- | --- | --- |
| 19 | Do you sometimes forget to take your medicine? | 1. No 2. Yes | | \|  \| \| --- \| |
| 20 | People sometimes miss taking their medicines for reasons other than forgetting. Thinking over the past 2 weeks, were there any days when you did not take your medicine? | 0 .No  1. Yes | | \|  \| \| --- \| |
| 21 | Have you ever cut back or stopped taking your medicine without telling your doctor because you felt worse when you took it? | 0 .No  1. Yes | | \|  \| \| --- \| |
| 22 | When you travel or leave home, do you sometimes forget to bring along your medicine? | 0 .No  1. Yes | | \|  \| \| --- \| |
| 23 | Did you take all your medicines yesterday? | 0 .No  1. Yes | | \|  \| \| --- \| |
| 24 | When you feel like your symptoms are under control, do you sometimes stop taking your medicine? | 0 .No  1. Yes | | \|  \| \| --- \| |
| 25 | Taking medicine every day is a real inconvenience for some people. Do you ever feel hassled about sticking to your treatment plan? | 0 .No  1. Yes | | \|  \| \| --- \| |
| 26 | How often do you have difficulty remembering to take all your medicine?  A. Never/rarely  B. Once in a while  C. Sometimes  D. Usually  E. All the time |  | | 1. = 0   B-E =1 |
|  |  | **Total score** | | \|  \| \| --- \| |
| Adherence Levels  1.Low adherence (>2)  2.Medium adherence (1 or2)  3.High adherence(0) | | | | |
| **Physiotherapy Services** | | |  | |
| 27. How many times have you defaulted on accessing physiotherapy services?  Specify_________________________ | | | \|  \| \| --- \| | |
